# Mu suppression – A good measure of the human mirror neuron system?

**DOI:** 10.1016/j.cortex.2016.03.019

**Published:** 2016-09

**Authors:** Hannah M. Hobson, Dorothy V.M. Bishop

**Affiliations:** Department of Experimental Psychology, University of Oxford, Oxford, UK

**Keywords:** Mirror neurons, EEG, Frequency, Mu suppression, Alpha, Beta, Methods, Baseline

## Abstract

Mu suppression has been proposed as a signature of the activity of the human mirror neuron system (MNS). However the mu frequency band (8–13 Hz) overlaps with the alpha frequency band, which is sensitive to attentional fluctuation, and thus mu suppression could potentially be confounded by changes in attentional engagement. The specific baseline against which mu suppression is assessed may be crucial, yet there is little consistency in how this is defined. We examined mu suppression in 61 typical adults, the largest mu suppression study so far conducted. We compared different methods of baselining, and examined activity at central and occipital electrodes, to both biological (hands) and non-biological (kaleidoscope) moving stimuli, to investigate the involvement of attention and alpha activity in mu suppression. We also examined changes in beta power, another candidate index of MNS engagement. We observed strong mu suppression restricted to central electrodes when participants performed hand movements, demonstrating that mu is indeed responsive to the activity of the motor cortex. However, when we looked for a similar signature of mu suppression to passively observed stimuli, the baselining method proved to be crucial. Selective suppression for biological versus non-biological stimuli was seen at central electrodes only when we used a within-trial baseline based on a static stimulus: this method greatly reduced trial-by-trial variation in the suppression measure compared with baselines based on blank trials presented in separate blocks. Even in this optimal condition, 16–21% of participants showed no mu suppression. Changes in beta power also did not match our predicted pattern for MNS engagement, and did not seem to offer a better measure than mu. Our conclusions are in contrast to those of a recent meta-analysis, which concluded that mu suppression is a valid means to examine mirror neuron activity. We argue that mu suppression can be used to index the human MNS, but the effect is weak and unreliable and easily confounded with alpha suppression.

## 1 Introduction

Since the discovery of “mirror neurons” in the macaque brain, researchers have investigated the presence of such neurons in humans, and considered what the functional role of the human mirror neuron system (MNS) might be. The human MNS has been posited to underpin action understanding, imitation, language and empathy, and has even been theorized to be the cause of an evolutionary leap in our ancestral history (Rizzolatti and Arbib, 1998, Rizzolatti and Craighero, 2004; see Baird, Scheffer, & Wilson, 2011 for a critical review of MNS involvement in empathy). MNS dysfunction has also been proposed to underlie the symptoms of autism spectrum disorders (Dapretto et al., 2006, Rizzolatti and Fabbri-Destro, 2010, Rizzolatti et al., 2009, Williams et al., 2001).

Mu suppression has been used to explore the MNS in both typical and autistic individuals. Mu is a range of electroencephalography (EEG) oscillations from 8 to 13 Hz, and is recorded from scalp electrodes corresponding to the sensorimotor regions of the brain (typically electrode sites C3, C1, Cz, C2, C4). When a person is at rest, the cells in the sensorimotor cortex fire in synchrony. When a person performs, observes or imagines themselves performing an action, the firing of these cells becomes desynchronised. This desynchronisation leads to reduced mu power, compared to when the cells were firing together (Pfurtscheller, Neuper, Andrew, & Edlinger, 1997). The key design feature of mu suppression studies is the comparison of an experimental condition to a baseline condition in which one would not expect the MNS to become active. If there is a reduction in mu power in the experimental condition compared to the baseline condition, the interpretation is that the experimental condition has activated neurons in sensorimotor cortex. Because mu suppression is seen both when an individual performs and observes an action, it has been taken as a proxy for the activity of the human MNS (Muthukumaraswamy and Johnson, 2004a, Muthukumaraswamy and Johnson, 2004b, Muthukumaraswamy et al., 2004, Oberman et al., 2007a, Oberman et al., 2007b, Pineda, 2005).

Such a relatively inexpensive and noninvasive technique for gauging the activity of the MNS in humans would greatly facilitate research on this system. However, not all researchers agree that mu suppression is a valid index of MNS activity (Aleksandrov & Tugin, 2012). Nevertheless, despite mixed findings of abnormal mu suppression in autism, some have suggested that mu suppression may be a viable target for neurofeedback therapy for individuals on the autistic spectrum (Pineda et al., 2008, Pineda et al., 2014). Indeed, mu suppression is rapidly becoming an established measure of mirror neuron activity that has been used to suggest roles for the MNS in processes such as in-group membership and empathy (Gutsell and Inzlicht, 2010, Moore et al., 2012).

One of the concerns raised in the literature surrounding mu suppression is whether it is reliably distinct from changes in alpha activity. Alpha activity was among the first EEG phenomena noted by pioneering electroencephalographer, Hans Berger, yet the precise function of alpha is still unknown. Alpha rhythms have been considered to reflect cortical idling (Pfurtscheller, Stancák, & Neuper, 1996), or the active inhibition of task-irrelevant processes (Klimesch, 1999). While the function of alpha activity is unclear, the reactivity of alpha is well documented. Alpha activity is functionally defined as “blocked or attenuated by attention, especially visual, and mental effort” (Niedermeyer & da Silva, 2005). Power in the alpha band is highest when a subject is awake with their eyes closed, and suppressed by mental effort, or drowsiness (Niedermeyer & da Silva, 2005). More difficult tasks elicit more alpha suppression (Gevins et al., 1997, Stipacek et al., 2003).

Mu is in the same frequency band as alpha (8–13 Hz), but alpha and mu are said to be distinguishable on the basis of topography and reactivity. Alpha activity arises in the posterior and occipital regions, while mu arises from the sensorimotor area. While changes in mu power are typically interpreted as being due to activity in the sensorimotor cortex, alpha power is thought to reflect attentional engagement (Klimesch, 1999, Pfurtscheller, 1992). Nonetheless, because of the overlap between mu and alpha activity, tight controls of attentional engagement should be a key feature of mu suppression experiments.

### 1.1 Evidence for alpha effects in mu suppression studies

Some authors have warned that mu suppression may be sensitive to activity from areas involved in visuomotor processes that are not considered to be part of the MNS (Braadbaart, Williams, & Waiter, 2013). Indeed, Perry and Bentin (2009) note that there was a relationship between alpha suppression recorded at posterior temporal sites and regional cerebral blood flow in the occipital lobes and BOLD signals in the parietal and visual cortices (Perry & Bentin, 2009). They caution that the desynchronisation of the 8–13 Hz frequency band might be due to the activity of many different networks, not just that associated with the MNS.

Other researchers have also voiced concern that changes in mu power may be being driven largely by attentional processes rather than mirror neuron activity. Aleksandrov and Tugin (2012) measured mu suppression during a large number of conditions, including conditions that contained no observation, execution or imagination of human movement, such as mental counting, or watching the movement of a non-biological object. Mu suppression during these conditions was not significantly less than the mu suppression seen in conditions where participants viewed human movement. Furthermore, they argued that tasks that were the most attentionally demanding produced the strongest mu suppression, and that mu suppression decreased over time, a finding they also attributed to attentional effects. Similar conclusions were reached by Perry and Bentin (2010). Because they found a similar pattern of changes in power at both occipital and central electrodes, they argued that the significant effect of condition may actually have been due to differences in attentional demands between their conditions, rather than differences in the activity of mirror neurons.

Indeed, Perry and Bentin (2010) cautioned that “mu suppression reports should always include not only experimental effects at the central sites, but also the occipital regions to help fully understand the phenomenon being studied.” (p1054). Where previous mu suppression studies have considered activity at occipital electrodes, findings have been mixed. Ruysschaert, Warreyn, Wiersema, Oostra, and Roeyers (2014) investigated changes in the alpha frequency band at occipital sites, but only during their imitation condition (when participants actively copied the movement they saw), not during their observation condition. Thus, while mu suppression during actual movement was specific to the central electrodes, it is unclear whether this was also true for this study's observation condition. Tangwiriyasakul, Verhagen, van Putten, and Rutten (2013) argued that the correlation between central and occipital electrodes was weak, suggesting that their results had not been affected by alpha. However, the correlation between C4 and O2 was .49 (*p* < .001), a not insignificant correlation. Lepage and colleagues also entered activity from electrode Oz in their analysis, and found that 8–13 Hz power at this site was significantly reduced during observe and imagine conditions (Lepage, Saint-Amour, & Théoret, 2008). Other papers have reported that other than C3, Cz, and C4, no other electrodes showed a consistent pattern of suppression (Bernier et al., 2013, Bernier et al., 2007, Oberman et al., 2005, Oberman et al., 2008). Thus, it seems unclear to what extent changes in mu power at the central electrodes are reliably distinct from changes in power at the occipital electrodes, regions more strongly associated with alpha. Finally, a recent study by Dumas and colleagues suggests that apparent mu suppression deficits in autism are not related to the MNS, but rather to alpha (Dumas, Soussignan, Hugueville, Martinerie, & Nadel, 2014). Dumas et al. analysed alpha-band activity over the whole scalp, and found that central mu suppression was preserved in their autistic subjects. Instead, alpha-band activity in other areas was abnormal. Even in their typical participants, suppression in the 8–13 Hz frequency band during action observation was significant over the whole scalp, but more strongly over the occipito-parietal region.

Overall, while concurrent functional magnetic resonance imaging (fMRI) studies suggest that mu suppression may represent activity in areas considered part of the MNS (Arnstein et al., 2011, Braadbaart et al., 2013, Mizuhara, 2012, Perry and Bentin, 2009), other processes that are not observation-execution matching also influence changes in mu power. This casts doubt on previous conclusions reached using mu suppression as an index of mirror neuron activity, particularly on higher level sociocognitive processes where the potential effects of attention may not be immediately obvious. For example, a recent mu suppression study argued that their results showed that the MNS is less responsive to outgroups and most responsive to people from your own group, which holds implications for empathy and prejudice (Gutsell & Inzlicht, 2010). The authors themselves note that different levels of mu suppression for different groups may be driven by attention, in that prejudice might bias attention against outgroups, thereby reducing the activation of the MNS. We would go one step further, and suggest that there is no need to appeal to the MNS as an explanation for these results – if there is an attentional bias towards one's own race then we can reasonably predict differing amounts of alpha suppression towards different groups. One way of controlling for potential attentional effects is to compare mu suppression to stimuli that are matched in their postulated engagement of the MNS. For example, Muthukumaraswamy et al. (2004) had participants view a hand interacting with an object versus a hand interacting with itself. Consistent with the predictions from non-human animal work on mirror neurons, they showed greater mu suppression in the former case (Muthukumaraswamy et al., 2004). We cannot, of course, rule out the possibility that a hand interacting with an object is more attentionally engaging, though participant ratings could be used to test this idea. In sum, as mu suppression is becoming a more mainstream method to measure the activity of the MNS, researchers must control for the possibility that attention (and thus alpha) may influence their results.

### 1.2 Choice of baseline in mu suppression experiments

The potential confound of attentional engagement assumes particular importance when considering the range of methods of calculating mu suppression that have been used in previous work. Mu suppression involves comparing power in the mu frequency band during an experimental condition to a baseline. Some researchers have opted to compare the power in their experimental conditions to a single baseline period, some have used an equivalent number of trials of a control condition, while others have baselined each individual trial separately.

Previous whole baseline conditions have included sitting quietly without stimulation, or visual white noise, or a motion control (e.g., Oberman et al., 2005). Clearly, in studies where participants have been asked sit and watch either no videos or videos that are not very engaging for long periods of time, it is feasible that the level of alpha activity would increase, due to attentional disengagement. For example, one study presented videos of visual white noise and bouncing balls that were 80 sec long (Oberman et al., 2005). Because alpha and mu waves are in the same frequency band (8–13 Hz), this could lead to an inflated ratio between the baseline and experimental conditions, leading to greater mu suppression. Some of these papers attempted to control for alpha by not including the first and last 10 sec of a stimulus in their analysis, the assumption being that any confounds caused by alpha will take place in these periods, due to the attentional effects of a stimulus initiating or ending (Oberman et al., 2005, Oberman et al., 2008).

Other groups have used a baseline of 1 sec prior to the onset of each trial as their comparison, either using a fixation cross, or presenting the first frame of the video as a static frame (e.g., Kumar et al., 2013, Muthukumaraswamy et al., 2006). This design is good for removing effects of long-term shifts in the EEG, for instance due to sweating over the time course of the experiment. By baselining each trial individually, such shift is accounted for, and the attentional effects induced by long baseline conditions are likely to be reduced, and inflated apparent mu suppression is less likely. Nevertheless, it could be argued that the onset of a moving stimulus would immediately engage attention more than a static image.

The issue of what baseline to use in mu suppression experiments was examined by Tangwiriyasakul et al. (2013). They recorded EEG data from 18 subjects, investigating what baselines may be ideal for obtaining maximal mu suppression. Their baselines included active and static stimuli, including bouncing balls, slowly moving flowers, static hand images and white stripes on a black screen. No optimal baseline for the whole group emerged – rather, different participants seemed to show bigger mu suppression effects for different baselines. The authors conclude that these findings suggest that calibration may be necessary for motor imagery experiments, in order to identify which baseline is optimal for the individual participant. However, these findings also suggest that mu suppression is not a reliable phenomenon. Indeed, the paper also reports that four of their participants did not show any mu rhythms in any of the five baseline conditions, and two showed mu, but showed no suppression. Thus, mu suppression, with any baseline, was only found for two thirds of their sample. Furthermore, reports from their participants suggest that attentional engagement could have played a role in these results:

“…many reported that during the BW [white stripes on a black screen] baseline it was difficult to maintain attention. Some of them started counting the white stripes on the screen… During the FL [flower] baseline, most subjects felt most comfortable and most relaxed; sometimes they lost their attention… During the dynamic baselines (BB and 2B) [bouncing ball conditions], some subjects said that they usually kept their attention to the ball(s).” (Tangwiriyasakul et al., 2013, p7).

### 1.3 Beta activity and the MNS

The convention of many mu suppression studies, particularly those focused on autistic individuals, is to define “mu” as activity in the alpha range (8–13 Hz). However, the rolandic mu rhythm consists of two spectral peaks, and gets its arch-like appearance from the dual contribution of alpha and beta range activity (Niedermeyer & da Silva, 2005). Thus, it is important to acknowledge not only the contributions of alpha but also beta activity in the previous findings in the mu suppression literature.

The beta frequency band is usually defined as 13–35 Hz, with a typical peak frequency of ∼20 Hz (Niedermeyer & da Silva, 2005). Beta activity is historically associated with sensorimotor behaviour (although recently it has been suggested that the role of beta in cognitive and attentional processes has been overlooked; see Engel & Fries, 2010 and Gola, Magnuski, Szumska, & Wróbel, 2013). Studies that have looked at both frequency bands suggest that while “rolandic alpha” (mu rhythm) is linked predominantly to the somatosensory system and somatosensory cortex, beta suppression is more related to motor processing and the primary motor cortex (Hari and Salmelin, 1997, Ritter et al., 2009). Like mu, beta activity is suppressed by voluntary movements, motor imagery and the observation of movements (Babiloni et al., 2002, Hari and Forss, 1998, McFarland et al., 2000), and changes in beta activity have also been suggested to index mirror neuron activity (Muthukumaraswamy and Singh, 2008, Rossi et al., 2002).

One evident risk in this field is that by focussing on one frequency band, we might miss key phenomena of interest. We have focused here on alpha and beta frequency ranges, but studies vary in terms of the precise frequency ranges used to define these, and indeed some argue for finer subdivision of these frequency bands (e.g., Pfurtscheller, Neuper, & Krausz, 2000). This, however, carries the complementary risk that if the choice of frequency band is open-ended, this provides ‘researcher degrees of freedom’ in post hoc analysis (Simmons, Nelson, & Simonsohn, 2011). To justify distinguishing different frequency ranges, we need studies that distinguish these a priori and consider whether there are reproducible differences in pattern of results between these.

### 1.4 Aims of this study

The aim of this study was to examine the validity of mu suppression as a measure of the human MNS, particularly in relation to whether conventional mu suppression designs are confounded with changes in alpha activity and attentional engagement, and also to explore whether the reactivity of beta follows the same pattern as mu.

Consistent with previous studies, we used videos of hand movements to elicit mu suppression. We also included a control stimulus that would not elicit mirror neuron activity, but which would be as engaging as the biological movement condition. For this, we chose kaleidoscope stimuli.

We examined whether changes in 8–13 Hz power at the central electrodes are distinct from changes in power at this frequency in the occipital regions, and whether high occipital alpha during baseline tasks could be a confounding factor in previous mu suppression designs.

Finally, we considered three different baselines that previous researchers have used to analyse their mu suppression experiments, and investigate how they might influence the results. These three baselines included long and short rest periods, and a static period at the start of each stimulus. We hypothesised that a long baseline condition as opposed to brief or trial-by-trial baselines inflates apparent mu suppression.

We considered how far the results from each of the three baselining methods showed the pattern that is predicted to be a signature of mu suppression, namely an interaction between condition and electrode site, such that the difference in mu suppression (8–13 Hz) between hand versus kaleidoscope stimuli is greater at the central than the occipital sites.

Subsidiary predictions were that mu suppression would be greater for the hand-with-object versus hand-no-object condition, and that the same overall pattern of activity would be seen for the beta frequency (13–35 Hz) as for the mu frequency.

## 2 Method

### 2.1 Participants

Our sample was 61 typical adult participants (see Appendix A: Power Analysis for sample size justification). Participants were recruited largely through the university's research participation scheme, and through poster and email advertisements. Our final sample included 19 males and 42 females, with a mean age of 22 years (18–33 years). Our sample included 51 right-handed participants, nine left-handed participants and one ambidextrous participant. The participants had no known neurological disorders, nor any diagnoses of autism spectrum conditions. Participants were required not to consume alcohol, or take any psychotropic medication, or any drugs likely to cause drowsiness, for the 8 h prior to the experiment.

### 2.2 Stimuli

Previous researchers have used a variety of stimuli to test mu suppression to human movement, including: hands grasping a manipulandum (Bernier et al., 2007, Muthukumaraswamy et al., 2004), hands manipulating chess pieces (Cheng et al., 2008, Fan et al., 2010), a hand opening and closing with no object (Oberman et al., 2005, Raymaekers et al., 2009), mouths sucking or biting, with or without a straw object (Muthukumaraswamy et al., 2006), a hand rotating a coin, or a coin being passed back and forth between two hands (Aleksandrov & Tugin, 2012). Our own stimuli were of a hand manipulating a pencil, or performing the exact same manipulative movements but without the pencil. While these stimuli are novel, the features of the stimuli map closely to those previously used in other mu suppression studies. We opted to include both object-based and non-object based stimuli in our experiment, as previous literature has argued that the presence of an object yields stronger mu suppression (Muthukumaraswamy et al., 2004). It was reasoned that reproducing the object effect would help ensure that our methods and findings are in keeping with and generalizable to other work.

We also included a control stimulus which should not activate the MNS. Selecting such a stimulus is far from straightforward, as movements of robotic hands have been found to activate mirror neuron areas (Gazzola, Rizzolatti, Wicker, & Keysers, 2007, but see Tai, Scherfler, Brooks, Sawamoto, & Castiello, 2004), and it has been argued that musical notation can produce significant mu suppression in musicians because of the associations between the sheet music and the movements required to play them (Behmer & Jantzen, 2011). Even stimuli of flowers opening, as used by Tangwiriyasakul et al. (2013), might be argued to be imitable (i.e., you could imagine opening a closed hand to produce a movement that was superficially similar). For these reasons, we chose to use black and white kaleidoscope videos as control stimuli. If significant mu suppression is seen during the observation of these stimuli it casts serious doubt on the validity of mu suppression as a pure measure of the MNS. Nonetheless, we also asked participants in a post-EEG questionnaire whether they could imagine themselves performing the actions in the videos (see Section 2.2.2 and Appendix C).

Equal numbers of trials of videos using the right and the left hand were shown to the participants. Our videos can be viewed on the Open Science Framework, under the project title “Mu suppression – a good measure of the human mirror neuron system?” (https://osf.io/yajkz/). Screenshots and further details concerning our stimuli can be found in Appendix B.

#### 2.2.1 Positive control

One of the key characteristics of mu suppression is that it occurs both when a participant observes and performs actions. Not all previous mu suppression investigations have included a movement condition. However, given it is this feature – activation during both observation and execution of movements – that has led researchers to propose it as a signature of mirror neuron activity, this investigation included a movement condition, based on a condition used in previous research that successfully elicited mu suppression (Woodruff, Martin, & Bilyk, 2011). This own movement condition acted as an outcome-neutral positive control condition.

#### 2.2.2 Subjective rating of engagement with stimuli

To test the hypothesis that previous differences between control and experimental conditions in mu suppression studies are driven in part by differing levels of engagement, we also asked our participants to rate their subjective levels of engagement in the different conditions. A copy of the post-EEG questionnaire can be found in Appendix C. We reasoned that if our analysis suggested that apparent mu suppression was being driven by changes in alpha and attentional effects, it would be expected that the pattern of mu suppression seen in the various conditions will follow the same pattern of subjective rating of attentional engagement. This questionnaire also allowed us to check whether participants could imagine themselves performing the “non-imitable” videos, the kaleidoscope patterns, and that they attended to the stimuli sufficiently (see Section 2.3.2).

### 2.3 Procedure

The study received approval from the ethics committee at the University of Oxford (Medical Sciences Interdisciplinary Research Ethics Committee Code: C1-2013-190). After reading the information sheet and signing the informed consent form, participants underwent the EEG. Participants were sat in a quiet room, and watched the stimuli presented to them via a laptop screen. There were three types of EEG condition: a) observing, b) resting and c) moving, based on the conditions used in previous research. In the observing conditions, participants watched the videos of the hand movements and kaleidoscope patterns. During the resting condition, participants were asked to sit quietly but to keep looking at the laptop screen, and not to close their eyes. The EEG conditions and trial types are summarised in Table 1.

#### 2.3.1 Timings

For each of the observing conditions (hand manipulating pencil, hand with no pencil, kaleidoscope patterns), there were 40 trials. In each video, the first 4 sec was a static picture of the hand/kaleidoscope patterns, which served as a baseline (see Section 2.6 Analysis plan). These 4 sec were followed by 2 sec of movement, and then 2 sec of a static final frame. The 2 sec of movement per trial means that each video condition had up to 80 sec of recording while participants observed the moving videos. The observing conditions were closely modelled on previous work (e.g., Muthukumaraswamy et al., 2006).

The resting condition was modelled on Bernier et al. (2007); participants were asked to sit quietly in front of a blank screen. A single long rest interval of 80 sec was included in each session, as well as short rest intervals of 8 sec each, interspersed within blocks of other stimuli.

For the own movement condition, participants were asked to tap their index finger and thumb together at a steady pace for 40 sec. This was done four times with each hand. Previous mu suppression research has used this movement to elicit mu suppression (Woodruff et al., 2011). The experimenter was able to watch the participant through a tinted window to ensure that they performed the finger tapping action.

Video stimuli were shown in eight blocks of 15, with videos playing back to back, except for five short rest trials (blank screen) included within each video block. Trials within each block were presented in a semi-random order. The order was constrained, such that a rest trial could not follow another rest trial (to ensure all short rest periods are 8 sec long, not 16 sec). The video/rest blocks were interleaved with the movement trials, such that participants watched 2 min of videos (with five short rest trials), then performed 40 sec of the finger tapping movement, then watched 2 min of videos, and so on. This interleaving of trials was intended to keep participants alert during the EEG.

The position of the long resting condition was counterbalanced across participants to occur at one of four places in the experiment – at the beginning, after two blocks of videos, after four blocks of videos, or after all eight blocks of videos.

#### 2.3.2 Measure of attention

In order to confirm that all participants included in the final analysis viewed and attended the stimuli properly, we included a coarse behavioural measure of attention. Previous studies into mu suppression have used continuous performance tasks as a means of ensuring their participants attended the stimuli (Oberman et al., 2007b, Oberman et al., 2008). These tasks have typically taken the form of counting a particular event. However, as noted above, alpha activity is known to be affected by mental activity (indeed, previous investigations of alpha have utilised counting targets as a task – see Klimesch, Doppelmayr, Russegger, Pachinger, & Schwaiger, 1998). Therefore, a sufficiently “light” cognitive task is required, so as not to influence the EEG. In our study, participants were told prior to the EEG recording that they would be asked questions about what they saw during the experiment at the end. During the EEG recording, three grey stars and three grey arrows appeared on the screen, following or preceding videos or rest periods, but never interrupting them. The stimuli were presented for 1 sec each time. Following the recording, participants were asked if they noticed anything during the experiment that was not a video of hands or kaleidoscope patterns. Participants who failed to report any of these extra stimuli, or inaccurately reported how many times these stimuli appeared were considered not to have attended to the stimuli properly, and were excluded from the analyses. While this is arguably a coarse measure of attention, it was reasoned that this task would motivate participants to attend to the stimuli properly, and identify any participants who were unable to do so.

### 2.4 Electrophysiological recording

EEG data were collected from 36 electrodes embedded in a cap using the 10–20 method of electrode placement, including four electro-oculograms (above and below the right eye, and to the sides of outer corners of each), and two electrodes on the mastoids. Electrolytic gel was applied at each electrode site to reduce the impedance of the electrode–skin contact. The impedance on all electrodes was measured and confirmed to be less than 40 KΩ both before and after testing. Recording was made at a sampling rate of 1000 Hz. The EEG data was recorded using a Neuroscan Nuamps system, and analysed using EEGLAB (Delorme & Makeig, 2004). All recordings were continuous, with no filters applied at the recording stage. Markers identifying the trial type were recorded at the start of the trial for each video and short rest trial, every 8 sec in the own movement condition, and every 2 sec during the long rest period. This allowed us to extract a similar number of 2 sec intervals from the long rest period as for each of the movement portions of the observing conditions.

#### 2.4.1 Electromyography

Viewing hand movements could lead to some automatic imitation, even if participants are instructed to remain as still as possible. In order to identify and exclude rest or observation trials in which participants generated muscle activity, we recorded an electromyogram (EMG) from the extensor digitorum communis (the arm muscle that extends the fingers). We recorded from these muscles on both the left and the right arm, using disposable ECG electrodes, at a sampling rate of 1000 Hz. The EMG data was recorded as additional channels in our EEG dataset and made bipolar in our analysis script. Details on how the EMG data was used to exclude movement trials can be found in Section 2.6.

### 2.5 Current source density (CSD)

EEG data was transformed to a “reference-free” format using CSD transformations. CSD estimates are second spatial derivatives of recorded field potentials (see Kayser & Tenke, 2005 for more details on CSD). CSD is essentially a spatial filter that minimises the problem of volume conduction, providing more accurate topographical results.

### 2.6 Analysis plan

Analysis was conducted using the following steps, using EEGLAB version 6.1 run in MATLAB. The script for analysing the data is available on Open Science Framework (https://osf.io/yajkz/).

Using this script, the continuous file was first epoched into segments starting at onset of the trial marker (0 sec) and lasting for 7 sec. All trials were baselined to be centred on an average of zero. Trials containing extreme values (greater than 350 μV) other than eye channels or frontopolar channels were removed. This is a much more extreme cut-off than is usually used because the goal at this point was just to remove trials with excessive movement artefact, but not to remove blinks.

We then removed any observation or rest trials in which the EMG activity recorded from the electrodes is above an individualised threshold. A non-active EMG was subtracted from the EMG recorded from the extensor digitorum communis to create a bipolar channel. The EMG activity in the own movement conditions was converted to root mean square values across all own movement trials, separately for the left and right arm. A threshold of 1.5 standard deviations below this average was used to remove trials in the rest or observation conditions that show muscle activity greater than this value.

The bipolar eye channels were subtracted to give one channel for vertical eye movements and another for horizontal eye movements. Data were then subjected to independent component analysis using single-order blind identification (see Bishop, Hardiman, & Barry, 2011). This was achieved by transforming the weight matrix for components into z-scores across all electrodes, and identifying those that have a z-score greater than 4.0. This is an arbitrary large value which has been determined in previous studies to identify signals due to blinks or to other artefact. Components whose activity is heavily focused on a single electrode were then subtracted from the signal.

To be included in the final analysis, a minimum of 16 trials per condition were required, after bad trials were rejected. Following the rejection of bad epochs, the remaining data were re-referenced offline to a CSD derivation using a CSD MATLAB Toolbox (Kayser and Tenke, 2006a, Kayser and Tenke, 2006b). The functions in the Toolbox were utilised by our analyses scripts. The Toolbox is freely available here: http://psychophysiology.cpmc.columbia.edu/software/CSDtoolbox/index.html.

The analysis was restricted to the sensorimotor and occipital electrodes C3, Cz, C4, O1, Oz and O3. Three methods for estimating mu suppression were compared, where the period from 2 sec to 4 sec post-trial onset is described as the early interval, and the period from 4 to 6 sec post-trial onset as the late interval. Note that these terms correspond to the static and active portions of the trials where hand stimuli are used. A frequency decomposition was conducted using the EEGLAB “spectopo” function, separately for early and late intervals for each of the six conditions: (a) Hand No Object, (b) Hand with Object, (c) Kaleidoscope patterns, (d) Short fixed stimulus; (e) Long fixed stimulus (f) Own Movement. Mean log power in the frequency range 8–13 Hz is defined as 10*log_10_(μv^2^/f), where f is frequency in Hz. The three methods are as follows:

*Method 1. Within-trial baseline*: Mean log power in the early interval was subtracted from mean log power in the late interval for all three observe conditions.

*Method 2. Between-trial baseline:* Mean log power in the late interval for the short rest trials was subtracted from that in the late interval for trials with hand or kaleidoscope stimuli, and own movement condition.

*Method 3. Single long baseline:* Mean log power in the long rest period was subtracted from that in the late interval for trials with hand or kaleidoscope stimuli, and own movement condition. In addition, as a further control, log power in the long rest period was subtracted from mean log power in the late interval for the short fixed stimuli trials: a contrast where no mu suppression should be observed.

**Fig. 1:**
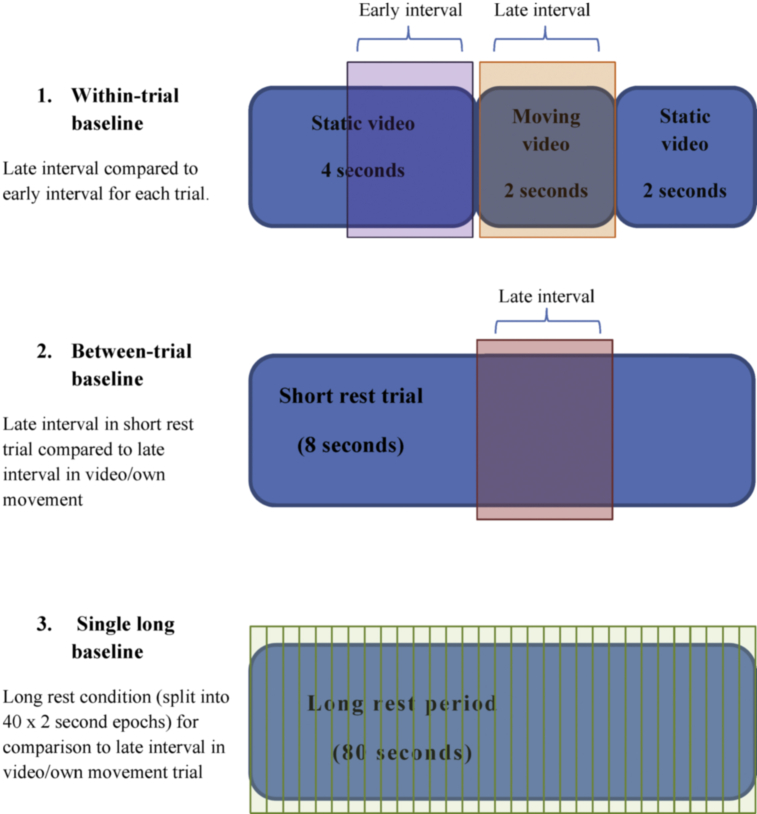
A diagram depicting the analysis using the three different baselining techniques. The period of the video in which the hand/kaleidoscope pattern moves (the orange section) is compared against one of three baselines: 1. The 2 sec early interval period immediately preceding the video when there is a static picture presented (purple); 2. The average power of the late interval period during the short (8 sec) rest trials (red); 3. The average power of the late interval periods in the long rest condition (green). The long rest period is composed of 40 × 2 sec epochs. In each 40 sec own movement trial, there are five triggers every 8 sec used to divide the movement trials up into five epochs. The own movement trials are analysed the same way as the video trials, comparing the power in the late interval the late interval in the short rest trials and the average power in the long rest condition.

Fig. 1 is a diagram depicting the three baselining methods.

For our main analysis, we conducted three 2-way repeated measure ANOVAs, for the three different baselining methods (short rest trials, long rest trials, and trial-by-trial baselines). In each analysis, the first factor is condition and the second factor is site (central and occipital). For the comparisons with rest trial baselines, all four conditions (hand no object, hand with object, kaleidoscope patterns and own movement) were compared. For the trial-by-trial baseline, the own movement condition is excluded, since the same movement is executed continuously. Electrodes C3, Cz and C4 are averaged together, as are electrodes O1, Oz and O3.

Results were analysed using repeated-measures ANOVAs rather than paired comparisons, so that we could test specific interactions between condition and electrode site. Because the three baselining methods are not independent, no direct comparisons were made between them. Rather, we considered how far any of the three methods showed the pattern of results that is predicted to be a signature of mu suppression, namely: on ANOVA, an interaction between condition and electrode site should be seen, such that the difference in suppression between hand-with-object versus kaleidoscope stimuli is greater at the central than the occipital sites.

In addition, we predicted that in the positive control condition (own hand movements) significant suppression of 8–13 Hz power would be seen at central sites (tested using one-sample *t*-test to compare observed power change to zero). Predictions about the hand-no-object observation condition are less clear-cut. The early mirror neuron theory focused on grasping of objects, and would not necessarily predict any MNS activity for these stimuli, but subsequent studies of mu suppression suggest it can occur with no object (e.g., Cochin and Barthelemy, 1999, Muthukumaraswamy et al., 2004). Following Muthkurawasamy et al. (2004) we predicted an interaction such that mu suppression would be greater for the hand-with-object versus hand-no-object condition. Finally, we predicted that the same overall pattern of activity across baselines and stimuli would be seen for the beta frequency (13–35 Hz) as for the mu frequency.

In order to limit the chance of Type I error, we pre-selected electrode sites (C3, Cz, C4, O1, Oz and O3).

If suppression of 8–13 Hz activity is seen to hand stimuli, but with a similar pattern of results for the central and occipital sites, this would suggest that differences between conditions could be accounted for by changes in alpha activity associated with attentional changes. In this case, we planned to use the results from the engagement questionnaire as a covariate to see if this could account for these results.

## 3 Results

### 3.1 Excluded participants

In total, 109 participants were recruited during the course of the study. Twenty seven participants were excluded for failing the attention check task. A further 13 participants were excluded as reliable EMG signals could not be obtained; as the EMG recordings were used to retain or exclude trials we could only include participants with data from these channels. A further three were excluded due to poor very EEG recordings, and five recorded datasets were found to have had too many trials rejected by our analysis script, and were therefore replaced with new participants. In total, 48 participants were excluded. In our final sample of 61 participants, a high number of trials were retained for each condition, following automated rejection in our analysis script (see Table 2).

### 3.2 Post-recording questionnaire responses

Table 3 shows the responses to the questionnaire, given to participants after the EEG recording session. We had intended to use the results of the engagement questionnaire as a covariate, if results from the occipital and central sites were found to be the same. However, given that mu suppression in all baseline techniques was weakest for kaleidoscope videos, but this stimulus was rated the most engaging by participants, and given the dissociation between mu and alpha in this condition, this was not deemed appropriate. Analyses on the questionnaire responses can be found in section “Supplementary unregistered analyses”, in the Appendix D. These show that the kaleidoscope videos were rated as the most interesting stimulus (although the actual ratings of engagement were not dissimilar across the conditions).

### 3.3 Results for the single long baseline

For each baseline technique, a two-way ANOVA was run, followed by the six planned comparisons (hand-object *vs* kaleidoscope, hand-no object *vs* kaleidoscope, and hand-object *vs* hand-no object, both at the central and the occipital sites). Correction for multiple comparisons was not performed since comparisons were planned before the data was collected. The mean changes in mu/beta power and standard errors are shown in Table 4, Table 5.

**Fig. 2:**
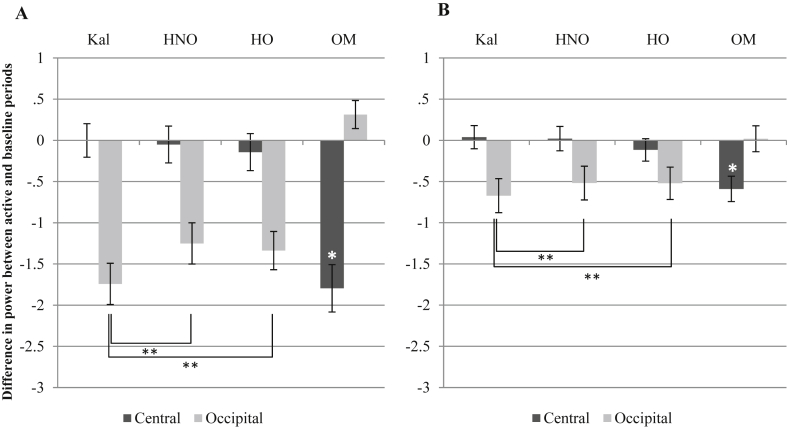
Graph A shows changes in the 8–13 Hz band (alpha/mu). Graph B shows changes in the 13–35 Hz (beta) band. Kal = kaleidoscope, HNO = Hand (no object), HO = Hand (with object), OM = own movement. Error bars are standard error. Planned comparisons between the video conditions that were significant are highlighted and asterisked: * indicates *p* < .05, ** indicates *p* < .01. Where one-sample *t*-tests found that suppression at central sites was significantly below 0, this is marked with a white *.

We first consider the results when the single long baseline condition was used to calculate mu/alpha (8–13 Hz) and beta (13–35 Hz) suppression. Fig. 2A and B shows the results using this baseline. For the mu band, there was a significant effect of site: *F* (1, 60) = 5.36, *p* = .024. Condition did not have a significant effect. There was also a significant interaction: *F* (1.36, 81.74) = 79.83, *p* < .001. Contrasts comparing suppression across the video conditions revealed that changes in the 8–13 Hz band were significantly different between the kaleidoscope and hand-object conditions at the occipital sites [*F* (1, 60) = 14.18, *p* < .001], but not at the central sites. Similarly, suppression during hand-no-object videos was significantly different from the kaleidoscope videos, in the occipital regions only [*F* (1, 60) = 15.17, *p* < .001]. Central mu suppression for hand-object and hand-no-object videos did not significantly differ. One-sample *t*-tests found that none of the video conditions had average suppression that was significantly lower than 0 at the central sites, however the own movement condition produced average mu suppression significantly below 0: *t* (60) = −6.25*, p* < .001.

For the beta band, there were no significant main effects of site or condition, but there was a significant interaction effect: *F* (2.02, 121.37) = 50.72, *p* < .001. Contrasts comparing suppression in the 13–35 Hz band across the video conditions revealed that suppression for kaleidoscope and hand-object videos at the occipital sites was significantly different [*F* (1, 60) = 7.05, *p* = .010], as was suppression for hand-no object and kaleidoscope videos at the occipital sites [*F* (1, 60) = 9.05, *p* = .004]. Hand-object and hand-no object videos did not significantly differ at either site. One-sample *t*-tests found that none of the video conditions had average suppression that was significantly different from 0 at the central sites, though suppression to own movement was: *t* (60) = −3.84, *p* < .001.^1^

Overall, with the long baseline, neither mu nor beta showed the pattern corresponding to the mirror neuron hypothesis. The only case where there was a selective suppression at central electrodes was when the participant engaged in hand movement. When observing hand movements, no suppression was seen. The occipital electrodes showed evidence of alpha suppression, which was greatest when observing the kaleidoscope patterns.

### 3.4 Results for the between-trial baseline

**Fig. 3:**
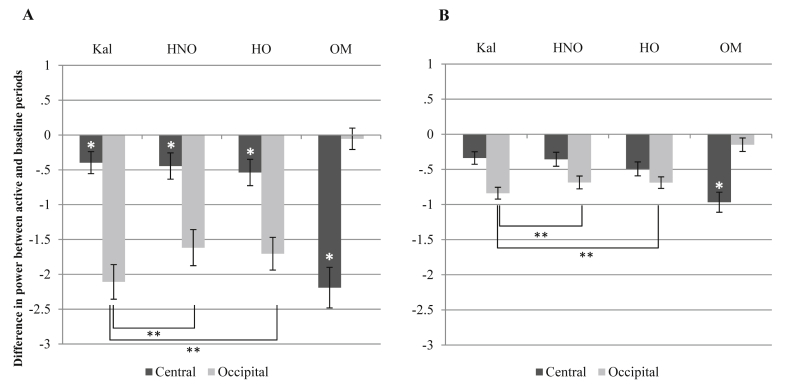
Graph A shows changes in the 8–13 Hz band (alpha/mu). Graph B shows changes in the 13–35 Hz (beta) band. Kal = kaleidoscope, HNO = Hand (no object), HO = Hand (with object), OM = own movement. Error bars are standard error. Planned comparisons between the video conditions that were significant are highlighted and asterisked: * indicates *p* < .05, ** indicates *p* < .01. Where one-sample *t*-tests found that suppression at central sites was significantly below 0, this is marked a white *.

The between-trial baseline was calculated by subtracting the average mu or beta power across the short rest trials from the active periods of the video conditions and own movement condition. Fig. 3A and B shows the results. Note that the pattern of differences between conditions will be the same as for the single long baseline analysis – this is because the same averages across the four conditions (the three video types and the own movement condition) are subtracted from a common average, this time based on the average power across the short rest periods. For mu, there was a significant main effect of site: *F* (1.60) = 8.34, *p* = .005. The effect of condition was not significant. There was a significant interaction: *F* (1.36, 81.72) = 79.83, *p* < .001. Contrasts comparing suppression across the video conditions showed that kaleidoscope and hand-object videos differed at the occipital sites only (*F* (1, 60) = 14.18, *p* < .001, the same as for the single long baseline). Similarly, suppression during hand-no object videos was significantly different from the kaleidoscope videos, at the occipital regions only [*F* (1, 60) = 15.17, *p* < .001]. Suppression to hand-object and hand-no object videos did not significantly differ at either site. On one-sample *t*-tests, mu suppression to the video stimuli was significantly below 0 for all three video conditions [For HO: *t* (60) = −2.85, *p* = .006; for HNO: *t* (60) = −2.36, *p* = .021; for kaleidoscope: *t* (60) = −2.51, *p* = .015]. The own movement condition also produced average mu suppression significantly below 0: *t* (60) = −7.52*, p* < .001.

For beta, there was no main effect of site, nor condition, but there was a significant interaction between site and condition: *F* (2.02, 121.37) = 50.72, *p* < .001. Contrasts comparing suppression across the video conditions for the 13–35 Hz band found that kaleidoscope and hand-object videos differed significantly at the occipital sites [*F* (1, 60) = 7.05, *p* = .010], as did hand-no object and kaleidoscope [*F* (1, 60) = 9.05, *p* = .004]. Hand-object and hand-no object videos did not significantly differ at either site. One-sample *t*-tests showed that suppression to all three video conditions were significantly below 0 at the central sites. [For HO: *t* (60) = −4.99, *p* < .001; for HNO: *t* (60) = −3.60, *p* = .001; for kaleidoscope: *t* (60) = −3.81, *p* < .001]. Own movement also resulted in beta suppression significantly below 0: *t* (60) = −6.85, *p* < .001.

In sum, with the between-trial baseline, the overall pattern of results was similar to that for the long baseline, except that there was evidence of suppression of both mu and beta at central sites. However, this suppression was no different for conditions observing hand movement than for the kaleidoscope condition, indicating it was not a reflection of mirror neuron activity.

### 3.5 Results for the within-trial baseline

**Fig. 4:**
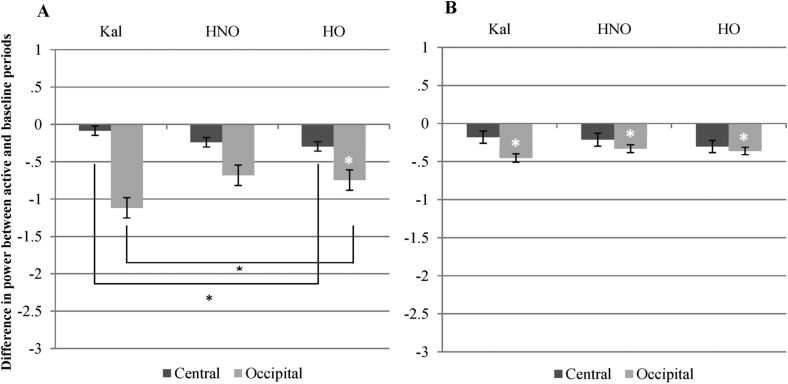
Graph A shows changes in the 8–13 Hz band (alpha/mu). Graph B shows changes in the 13–35 Hz (beta) band. Kal = kaleidoscope, HNO = Hand (no object), HO = Hand (with object). Error bars are standard error. Planned comparisons between the video conditions that were significant are highlighted and asterisked: * indicates *p* < .05. Where one-sample *t*-tests found that suppression at central sites was significantly below 0, this is marked with a white *.

This baseline was calculated by subtracting mu or beta power during the static image component of the videos from the active portion of the videos, on a trial-by-trial basis. Fig. 4A and B shows the results. (Note that standard error is smaller with this baseline, as the active periods of the videos are baselined with the static portions of the videos from the same condition, hence reducing effects of between condition variation). For mu, there was a main effect of site: *F* (1, 60) = 36.23, *p* < .001. There was no main effect of condition. There was also a significant interaction between condition and site: *F* (2, 120) = 12.93, *p* < .001. The planned contrasts revealed that the kaleidoscope and hand-object videos were significantly different both at the occipital [*F* (1, 60) = 11.54, *p* = .001] and central sites [*F* (1, 60) = 4.82, *p* = .032]. Hand-no object and kaleidoscope videos were significantly different at the occipital sites only: *F* (1, 60) = 16.27, *p* < .001. Hand-object and hand-no object videos did not significantly differ at either site. One-sample *t*-tests found that only the hand-object videos produced mu suppression that was significantly below 0: *t* (60) = −2.76, *p* = .008 (although there was trend for near significance for the hand-no object videos: *t* (60) = −1.97, *p* = .054).

For beta, there was a main effect of site: *F* (1, 60) = 8.154, *p* = .006. There was no main effect of condition, nor an interaction. None of the planned contrasts were significant. One-sample *t*-tests found that suppression for all three video conditions were significantly below 0. [For HO: *t* (60) = −3.74, *p* < .001; for HNO: *t* (60) = −2.50, *p* = .015; for kaleidoscope: *t* (60) = −2.24, *p* = .029].

To summarise, the within-trial condition was the only baseline to show the predicted pattern of mu suppression that would be consistent with mirror neuron activity. When observing a hand manipulating an object, there was significant mu suppression, whereas this was not seen when observing the kaleidoscope patterns: mu suppression differed significantly between these two conditions. As indicated in Fig. 4, observing a hand moving without an object showed a trend in the same direction as the hand with object, but this fell short of statistical significance. This pattern was not seen for the beta frequency band, where suppression at central sites was seen to all three types of stimuli, without any difference between conditions.

### 3.6 Short rest periods versus long rest period

Average mu power from the long rest condition was subtracted from the average power across the short rest periods, and the significance of this difference assessed using a one-sample *t*-test. This showed that both central mu and occipital alpha power were higher in the short rest periods than the long rest condition, and that the difference was significantly different from 0 [for central sites: *t* (60) = 3.01 *p* = .004; for occipital sites: *t* (60) = 5.42, *p* < .001].

### 3.7 Unregistered analyses – percentage of participants showing expected mu suppression effects

In addition to the analyses above, we considered how many of our participants showed expected mu suppression effects – that is, mu suppression significantly below 0 when performing and observing actions. The report by Tangwiriyasakul et al. (2013) suggested around a third of participants do not show predicted effects. We modelled this section of our analysis on their paper, in which they used *t*-tests to assess for each participant whether or not they demonstrated significant changes in mu for their different baseline techniques.

Thus, for each of our participants, we calculated a 95% confidence interval (CI) for the change in mu power at electrodes C3, Cz and C4 (the paper by Tangwiriyasakul et al. generally considered channels separately, so we did the same for parity), for the observation and own movement conditions. In order to be considered to have shown the expected mu suppression effect in a given condition, a participant was required to show a CI that did not cross zero (demonstrating mu suppression significantly below 0) for *at least one* electrode site. We also examined how many participants showed significant mu suppression during their own movement; Tangwiriyasakul et al. (2013) did not include an own movement condition, but as mu suppression is considered an index of motor cortex activation this provided a positive control. For this, we considered right hand and left hand movement conditions separately.

Table 6 shows the percentage of participants who *failed* to show the expected mu suppression effect at any of the three electrodes, for each hand video condition and own movement condition, by baselining technique. While not all participants showed significant mu suppression to their own movement, between a sixth and a third of participants failed to show the expected suppression effect when observing the hand videos. Consistent with the prior analyses, the greatest proportion showing mu suppression was for the hand-with-object condition using the within-trial baseline (note that it was not possible to baseline the own movement condition with this technique).

### 3.8 Summary of results

We outlined that a key condition for mu suppression to be considered a valid indicator of MNS activity would be observing an interaction between condition and electrode site, and that the difference in suppression between hand and kaleidoscope stimuli would be greatest at the central sites. Although significant site by condition interaction effects were seen for the 8–13 Hz band, these effects were not due to significantly stronger central suppression to biological videos – instead, these statistical interactions were due to stronger occipital suppression to kaleidoscope videos and strong central suppression to participants' own movements. For the hand videos, suppression was always stronger at the occipital sites. The only analysis providing evidence of specific central mu suppression to hand videos was that using the within-trial baseline. It would appear that the static-period (within trial) baseline represents a better baselining technique – this was the only baseline in which the planned comparisons found specific suppression for hand-object videos. Furthermore, a higher proportion of individual participants showed mu suppression effects when considering this baseline.

For the beta band, the only main effect of condition was for the beta band for the between-trial baseline (and even for this effect, hand-object and hand-no object videos did not differ from kaleidoscope videos at the central sites, only at the occipital). Similar to the mu-band, we failed to find evidence of a specific reaction of the beta band to hand videos.

## 4 Discussion

Rest periods are commonly used in mu suppression investigations as baseline conditions. However, using two different rest-baselining methods and examining changes in power at both the central and occipital sites, we failed to find evidence for specific mu suppression to videos of human movement. The final method, using a baseline measure from a static stimulus at the start of each trial, gave much less variability in measures of mu suppression (as indicated by the narrower CIs around the mean values for this baseline), and did give a pattern of results that was consistent with mirror neuron activity, although as found in previous research, this was much reduced compared to the mu suppression when performing movements (Woodruff & Maaske, 2010).

Our control conditions (watching kaleidoscope patterns, and performing finger tapping movements) show that it is possible to dissociate mu from occipital alpha. Our positive control condition, in which participants performed movements themselves, confirms that desynchronization of mu at the central sites captures the activity of the motor areas. Furthermore, in this condition, where no visual stimulus is observed, there was no alpha suppression at occipital sites, whereas alpha suppression was substantial when watching visual stimuli. Indeed, if mu suppression was simply a reflection of alpha confounding, and mu suppression was inherently tied to changes in attentional engagement, it would be predicted that the kaleidoscope videos, rated the most engaging by participants, would show both the strongest occipital and central suppression. Instead, while kaleidoscope videos yielded significantly stronger occipital alpha suppression than the biological videos, the difference between the hand and kaleidoscope videos at the central sites was non-significant, or in the opposite direction. This is an encouraging finding, as it suggests engagement and attentional issues are factors that can be separated from mu suppression, and should be considered and controlled in future mu suppression work.

Similar to Tangwiriyasakul et al. (2013) we found that a significant minority of our participants failed to show the expected suppression effect to hand videos, even in our optimal within-trial baseline condition. These participants were typical adults with no reported history of any neurological disorders, nor any diagnoses of autism spectrum conditions. These observations highlight that mu suppression to observing human action is not a universal finding, limiting its power as an experimental tool.

In some of our baseline techniques, we observed significant suppression at the central sites to videos of kaleidoscope patterns, stimuli we would not predict to activate the MNS. The question then arises as to whether participants might somehow have imagined themselves performing the movements they observed. This seems implausible. These stimuli are highly abstract, and were selected as stimuli that could not be easily embodied. Furthermore, participants were asked at the end of the recording if they felt they could perform or imitate the patterns, and almost all of our participants reported that they could not. Limited differences in central mu suppression between hand and kaleidoscope videos call in to question the specificity of mu suppression, and again weaken arguments that this is a valid measure of the MNS.

Broadly, our results are consistent with a recent meta-analysis of mu suppression studies (Fox et al., 2015), which was published during the data collection phase of this registered report. The current report included more participants than any of the studies included in their meta-analysis, and (unlike many of the investigations reviewed in the meta-analysis) is sufficiently powered. Fox et al. (2015) determined from the studies they reviewed that there is strong, central-specific suppression during action execution, no significant effect of biological (hand) versus non-biological (kaleidoscope) conditions on suppression during action observation, and a lack of central-specific effects during action observation – results similar to our findings for the first two of the baseline conditions. Interestingly however, despite these similarities, we have arrived at different conclusions. Fox et al. (2015) argue that mu suppression can indeed be used to index MNS activity. We, by contrast, argue that evidence for mu suppression is only apparent when a specific kind of within-trial baseline is adopted that controls for some extent for variability across a session. When other baselines are used – as was the case for many of the studies in the meta-analysis – the impression is that mu suppression is typically confounded with alpha suppression, which occurs in response to the presentation of a new visual stimulus.

Fox et al. (2015) did consider a number of moderating factors in their analysis, including type of baseline used, and found no moderation effects of baseline on the effect size of mu suppression. However, this is not in conflict with our argument that baseline is an important factor. Technically, the strongest mu suppression in our current study was observed when using a short rest baseline, but it is apparent that these results are confounded by alpha. Considering baseline's effect on the *strength* of mu suppression alone will not prove that this is indeed an important factor – we argued that *specificity* is important for determining mu suppression's validity. We opted to consider the pattern of significant and non-significant mu suppression across our conditions, and only the within-trial baseline showed a pattern of suppression that was specific to biological stimuli.

Given our results, we reason that treating mu suppression as a proxy for mirror neuron engagement, and using it as a basis for neurofeedback therapy, requires serious caution. While the original function of the MNS was purported to be action-understanding, theories about the human MNS have evolved radically to encompass potential roles in a number of social and communicative functions, including empathy (for a review of the MNS and empathy see Baird et al., 2011). Indeed, several investigations have used mu suppression in an individual-differences approach, as a gauge of the quality or responsiveness of an individual's mirroring system. This is then correlated with personal characteristics, such as empathy or prejudice (e.g., Cheng et al., 2008, Gutsell and Inzlicht, 2010). Our study cannot speak to whether the MNS is involved in such processes or not, but we do find worrisome the notion that such studies may be taken as evidence that mu suppression is a valid and reliable measure of the human MNS, especially as such studies would seem generally quite underpowered to examine these correlational questions, and corrections for multiple comparisons have not always been adhered to. In fact, a study by Silas, Levy, Nielsen, Slade, and Holmes (2010) which did use appropriate corrections concluded empathy measures were unrelated to individual differences in mu suppression.

We found that mu suppression is not consistently demonstrated from individual to individual (even in typical participants). One possibility is that individual variation in mu suppression is meaningful and related to some characteristic that we failed to measure. Nonetheless, we would caution that our study suggests that mu suppression is not specific to viewing biological stimuli (we argue a key characteristic of the MNS), and thus its use as a measure of the quality of an individual's MNS seems dubious. Furthermore, we are not aware of any data on reliability of mu suppression – that is, how variable is an individual's mu suppression within and between testing sessions? Correlating mu suppression with individual differences in empathy or prejudice would seem to imply it has some relatively fixed or stable quality to it.

We were able to look at one individual difference in relation to mu suppression, namely gender. Although the meta-analysis by Fox et al. noted that studies with predominantly male samples reported stronger effects, previous investigations studying gender differences in mu responses reported that females exhibit stronger mu desynchronisation to observation of biological movement (Cheng et al., 2008, Cheng et al., 2006, Silas et al., 2010). Our sample had a high proportion of females, so any sex difference in mu suppression could influence our results. Accordingly, we did a further unregistered analysis to explore this issue. We did not find any gender effects on activity at the central sites during action observation (See Appendix D: Supplementary unregistered analyses).

Inconsistent findings in relation to gender raise questions about correlations found with behavioural measures of individual differences. As Vul, Harris, Winkielman, and Pashler (2009) noted, correlations between behavioural and neurofunctional measures often overestimate effects: “Such an analysis will inflate observed across-subject correlations and can even produce significant measures out of pure noise.” (Vul et al., 2009, p279). Their article was concerned with fMRI studies, but it raises warnings about the dangers of studying individual differences using neurofunctional measures of unknown reliability. We recommend that any researchers investigating correlates of mu suppression should first establish the reliability of their measures.

As well as considering the validity of mu suppression as an index of MNS activity, we also considered a second frequency band, the beta band. Researchers have suggested that mu maybe be more related to sensory processing rather than motor activity, and changes in beta power, not mu, are indicative of motor cortex activity (Coll et al., 2015, Ritter et al., 2009). A recent meta-analysis of mu suppression studies called for further investigation of beta-band responses (Fox et al., 2015). Overall, as predicted, the pattern of results obtained for beta was similar to those obtained with the alpha/mu band. Our results suggest beta suppression is no better an index of mirror neuron activity than mu. However, other investigations have used post-movement beta rebound effects (rather than suppression during stimulus presentation) to examine beta's responses. Following medial-nerve stimulation, when beta typically “rebounds” to higher than pre-stimulation levels, showing participants videos of actions has been found to suppress this rebound effect (Muthukumaraswamy and Johnson, 2004a, Muthukumaraswamy and Johnson, 2004b). Reduced rebound suppression has also been noted in participants with autism (Honaga et al., 2010). Further work will need to be done to ascertain whether post-movement rebound effects offer a better measure of MNS engagement than simple suppression during stimulus presentation.

### 4.1 Controlling for attention and alpha effects

In their recent meta-analysis, Fox et al. (2015) discussed the problems of attentional effects and alpha on mu suppression investigations. They argued that mu suppression studies should include a condition in which no action is observed or executed, but in which participants experience the same attentional demands as the other experimental conditions. This attention condition could then be subtracted from experimental conditions to control for attentional confounds.

Although this recommendation is well-justified, in practice it is hard to implement because we do not have a way of matching attentional demands across tasks. Some previous investigations have used continuous performance tasks to ensure participants maintained attention to the screen, but this may be problematic when long resting baselines are used (when there are naturally no stimuli for participants to continuously count or monitor).

In the current investigation, we picked an attention check that was less demanding than a continuous performance task, which was selected to motivate participants to attend to the screen, and to provide a broad filter for those who failed to do so. It is noteworthy that a large number of participants (27 of 109) failed this attention check task and had to be replaced. Our final sample included only participants who passed this test, but the high rate of attrition does suggest that attentional engagement does need to be considered and sufficiently monitored or controlled for in mu suppression studies. Future work will need to strike a balance between demanding attention tasks (which could increase alpha suppression and lead to confounding), and ensuring that participants are paying sufficient attention to the stimuli they are observing.

We had predicted that the long rest period would inflate alpha levels in the baseline, and thus inflate apparent mu suppression. However, our results do not support this – significant mu suppression was not seen for any of the video conditions using this baseline. Indeed, stronger occipital alpha and central mu power was seen in between-trial baseline, which used short rest periods. This result is unexpected, as the stimulus the participants are seeing in the short and long rest periods is exactly the same. What this suggests is that the time-course of alpha and mu responses is also important. Sampling alpha/mu levels in the middle of the long rest condition is not the same as sampling them at the beginning or end of this period, and similarly sampling these levels when participants are viewing a blank screen but when they have just been viewing dynamic videos is not the same. It may be that over the length of the long resting baseline, alpha levels change, or that going from viewing a video to a blank screen may induce greater alpha enhancement than sitting without stimulation for a long period of time.

We found that the kaleidoscope videos produced significantly more suppression in the alpha and beta bands at the occipital regions, regardless of baseline. These stimuli were also rated by participants as the most engaging. These stimuli did differ from our hand videos in a number of ways, and potentially very slight differences in overall level of motion, or contrast, could have had an impact on the differences in alpha suppression between these video types. However, this finding does not explain why we failed to find an effect of video condition on central mu suppression, in two of our three baseline techniques.

Finally, one point to note is the suggestion made by Fox et al. (2015) that the tight association between alpha and mu might be a reflection of “a close coordination of action and attention”. (Fox et al., 2015, p6). While Fox et al. (2015) themselves do not elaborate much on this point, what their idea entails is that mu and alpha reflect separable but highly related processes, and to an extent seeing changes in alpha should not alarm us, as they may be an inherent part of action processes, alongside motor activity. It is an interesting notion. For this study however, if one accepts that during action attentional processes are highly probable and perhaps and natural part of action processes, why are the alpha and mu responses to participants' own movements so clearly distinct? Our data would have to suggest this close coordination only occurs for observing others' actions.

### 4.2 Object effects in mu suppression

Greater mu suppression to videos in which participants interact with an object has been found previously (Muthukumaraswamy and Johnson, 2004a, Muthukumaraswamy and Johnson, 2004b). We did not replicate this finding and the recent meta-analysis by Fox et al. (2015) also failed to find a significant moderator effect of object versus non-object-directed stimuli. Potentially, one reason previous reports may have found stronger mu suppression to transitive versus intransitive actions may have been more related to the presence of goals or discernible actions, as opposed to the mere presence of an object. In Muthukumaraswamy and Johnson's (2004a and 2004b) investigation, their stimuli involved precision grips made on an object, and precision-gripping movements made without contacting an object. A precision grip may be described a goal-oriented action, whereas in our stimuli the videos in which the hand interacts with a pencil are less clearly goal-based actions.

Another possible explanation for why in both our study and in the recent meta-analysis mu suppression object effects have not replicated could be that mu suppression is more related to sensory rather than motor stimuli, and that the tactility of stimuli affect the strength of mu suppression observed. Using a cross-modal repetition suppression design, a recent paper by Coll et al. (2015) showed that repetition effects were only found in conditions where the tactile components of the stimuli were repeated, not when the motor components were repeated. They thus concluded that mu suppression is more related to sensory rather than motor mirroring. Arguably, our own stimuli differed from Coll et al.'s in that there was quite minimal contact between the hand and object in the hand-object videos. Potentially this could mean that there was not enough of a tactile element to the videos to cause sufficient suppression and obtain a significant object effect.

### 4.3 Suggestions for going forward

We would not want to suggest that our procedure is “bullet-proof” or a “gold-standard” way of doing mu suppression studies. Instead, we hope that our study will serve as a platform for discussion around how best to conduct investigations going forward, so that researchers can converge upon a reliable setup that is most likely to provide solid ground for robust breakthroughs in understanding. Mu suppression studies are already widely used and cited. What do mu suppression studies in the future need to consider? Several important suggestions were outlined in the recent meta-analysis by Fox et al. (2015). These include the need for execution and observation conditions to be included in future studies, and ways to deal with potential alpha confounding. Here, we re-iterate salient points and add further suggestions of our own.

Our findings highlight the importance of considering and presenting the results from regions associated with alpha, outside of the central sensorimotor strip. As described earlier, a recent paper utilised a whole-brain approach to analysis to re-examine the issue of mu suppression deficits in autism (Dumas et al., 2014). When only examining the central electrodes, the previous reports of mu suppression abnormalities in autism were replicated. However, when their analysis widened to include other regions it was clear that the key sites of difference between control and autistic participants were not at the central sites, but rather in the frontal and occipital regions. Indeed, Fox et al. (2015) found in their meta-analysis that for action-observation conditions, effects were not specific to the central regions, and they noted that many studies failed to report findings from other sites. To be confident that mu suppression is indexing changes in activity in motor areas, it must be ruled out that these changes could be coming from elsewhere.

Another concern when reading mu suppression literature is that there seems to be much room for analytic flexibility, a factor known to be associated with poorer reproducibility (Ioannidis, 2005). For example, the parameters of the mu band are not fixed, and different studies use different definitions of the mu band, with some suggesting the mu-band needs to be further divided up (Pfurtscheller et al., 2000). Having agreement, discussion and transparency around how data collected from mu suppression studies is analysed will be important. Our analyses were based on what seemed to be the prevailing approach in the field, and accompanying this paper, we have made our analysis scripts open to the scientific community to download, use and adapt.

Finally, considering data at the individual level will be useful in ascertaining to what extent mu suppression to action observation is a reliable phenomenon, dependable enough for experimental or proposed clinical use. In common with some previous experiments, we have noted that mu suppression is not observed in a significant minority of typical participants. If mu suppression is to be continued to be used for inferring the processes mirroring systems are involved in, or comparing groups (such as autistic and typical participants), understanding why so many participants do not show expected mu suppression effects will be important. Studies of mu suppression in autism usually present data at the group level, comparing average changes in mu, but it would be intriguing to know whether the proportion of participants showing expected mu suppression effects differ between the groups - do more participants with autism show no mu suppression, or an indeed an increase in mu when observing actions?

## 5 Conclusions

We have conducted what we believe to be the largest mu suppression study to date, investigating mu suppression's validity as a measure of human MNS activity, and the importance of baseline methodology. Our results suggest that mu suppression calculated using resting baselines is not specific to biological stimuli, nor the central motor regions. Similar results were found for beta-band suppression. Using a baseline of a static image improved the specificity of mu responses, but even when this baseline technique was used, a significant minority of typical participants did not show the expected mu suppression effects. This has implications for the future use of mu suppression in experimental settings and for clinical applications.

### 5.1 Links to data and scripts

Analysis scripts, stimuli, links to our raw EEG files, and other details of our experiment can be found on the Open Science Framework, project name “Mu suppression – a good measure of the human MNS?” https://osf.io/yajkz/.

## Figures and Tables

**Appendix A1:** The power (1-β) of the planned statistical tests. For these calculations, the following parameters were used: *N* = 61, *ɛ* = 1/*m*−1 (where *m* is the number of measurements. For the main effect of condition, this is 4. For the interaction, this is 8. For the trial-by-trial baseline these are 3 and 6, respectively.), the correlation between measures = .6, and standard medium effect sizes (*f* = .25, *d* = .5).

Proposed statistical test	Power
Repeated measures ANOVA – main effect of condition	.94
Repeated measures ANOVA for trial-by-trial baseline – main effect of condition	.96
Repeated measures ANOVA – main effect of site	.99
Repeated measures ANOVA – interaction between site and condition	.90
Repeated measures ANOVA for trial-by-trial baseline – interaction between site and condition	.91
Paired *t*-tests (two-tailed)	.97

**Figure:**
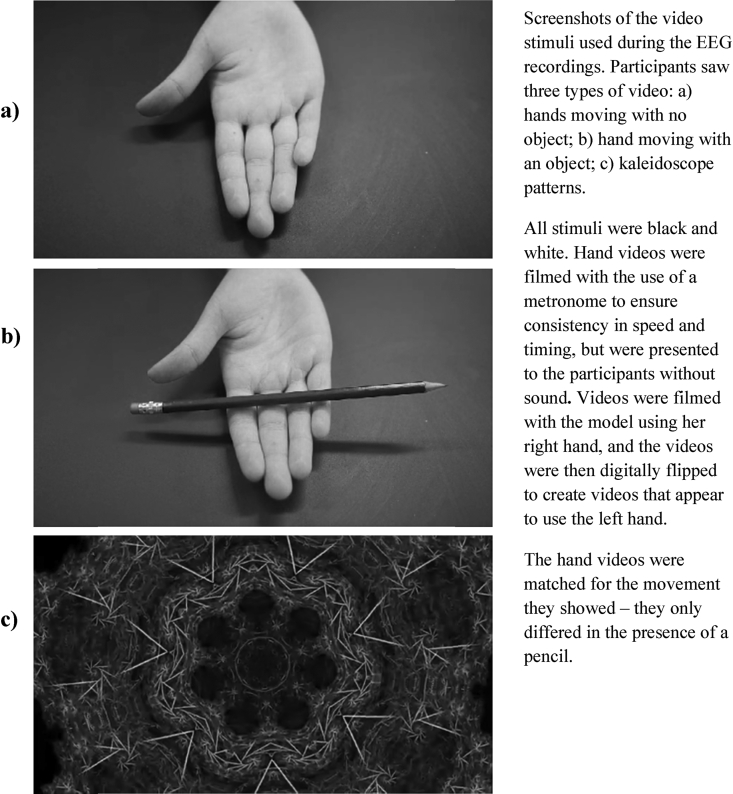


**Table 1:** The conditions during the EEG recording.

Condition	Trial type	Description
Observation condition	Hand action with object (HO)	8 sec videos, in which a hand interacts with an object (a pencil). 40 trials in total.
Hand action without object (HNO)	8 sec videos, in which a hand performs actions. There is no object in this video. 40 trials in total.
Kaleidoscope pattern (Kal)	8 sec videos of a kaleidoscope pattern. 40 trials in total.
Rest condition	Short rest baseline condition	8 sec period of a blank screen. Participants instructed not to move, just like in the video conditions. 40 trials in total.
Long rest baseline condition	80 sec period of a blank screen. Participants instructed not to move, just like in the video conditions. This condition is presented as one continuous trial and later epoched into 2 sec periods.
Own movement condition		40 sec period in which participants are instructed to tap their finger and thumb. Four 40 sec periods for the right hand, and four for the left hand (eight in total).

**Table 2:** Average number of trials retained per condition in final sample of 61 participants. Note that from the participants' perspective, there is only one long rest trial – the average presented here represents number of epochs retained.

Condition	Hand-no object	Hand with object	Kaleidoscope	Own movement	Short rest periods	Long rest periods
Mean no. trials retained	34.08	32.87	33.59	39.07	33.98	33.52

**Table 3:** Responses to the post-recording questionnaire. Numbers in parentheses represent standard deviation. When rating average engagement, participants were asked to rate on a scale of 1–5 with five being very engaged. When rating difficulty to perform, participants were asked to rate on a scale of 1–5 with five being very difficult to perform. The questionnaire can be found in Appendix 3.

	Kaleidoscope	Hand (no object)	Hand (with object)	Rest period
% Rated most interesting	65.6	6.6	26.2	1.6
% Rated least interesting	4.9	14.8	1.6	78.7
Average engagement	3.57 (1.02)	2.97 (.95)	3.39 (.95)	2.05 (1.04)
Average difficulty to perform	4.39 (1.05)	1.98 (1.11)	1.78 (1.02)	N/A
% Judged could imitate	11.5	96.7	95.1	N/A

**Table 4:** Mean changes in alpha-band (8–13 Hz) power for each condition, and baseline technique. Numbers in parentheses represent standard error.

	Kaleidoscope	HNO	HO	Own Movement
**Single-long baseline**				
Central	−.001 (.20)	−.050 (.22)	−.143 (.22)	−1.796 (.29)
Occipital	−1.742 (.25)	−1.250 (.25)	−1.337 (.23)	.313 (.17)
**Between-trials baseline**				
Central	−.397 (.16)	−.445 (.19)	−.538 (.19)	−2.191 (.29)
Occipital	−2.109 (.25)	−1.617 (.26)	−1.704 (.24)	−.054 (.15)
**Within-trials baseline**				
Central	−.085 (.11)	−.240 (.12)	−.295 (.11)	
Occipital	−1.118 (.14)	−.681 (.12)	−.745 (.11)	

**Table 5:** Mean changes in beta-band (13–35 Hz) power for each condition, and baseline technique. Numbers in parentheses represent standard error.

	Kaleidoscope	HNO	HO	Own Movement
**Single-long baseline**				
Central	.039 (.14)	.020 (.15)	−.116 (.14)	−.590 (.15)
Occipital	−.672 (.21)	−.519 (.20)	−.521 (.20)	.019 (.16)
**Between-trials baseline**				
Central	−.338 (.09)	−.356 (.10)	−.493 (.10)	−.968 (.14)
Occipital	−.839 (.08)	−.686 (.09)	−.689 (.08)	−.149 (.10)
**Within-trials baseline**				
Central	−.181 (.08)	−.213 (.09)	−.303 (.08)	
Occipital	−.453 (.05)	−.331 (.05)	−.362 (.05)	

**Table 6:** The percentage of participants who do not show expected mu suppression effects at any of the central electrode sites (C3, Cz and C4), for the video conditions and the own movement conditions (shown with the right and left hand separately). A given participant was considered to have shown mu suppression if the 95% CI around the average difference in mu between the active period and baseline period did not cross zero, for any of the three electrode sites.

		Video condition		Own movement condition	
HNO	HO	R	L
Baseline technique	Within-trial baseline	21.3	16.4	N/A	N/A
Between-trial baseline	21.3	21.3	8.2	4.9
Single long baseline	24.6	29.5	4.9	3.3

## Appendix A Power analysis (submitted during registration of report)

Previous effect sizes have not been routinely reported in mu suppression literature. This makes selecting an adequate effect size for the basis of a power analysis difficult. However, if mu suppression is a valid biomarker for social cognitive processes, or indeed autism (Cheng et al., 2006, Perkins et al., 2010), and a potential target for neurofeedback therapy (Pineda, Juavinett, & Datko, 2012), then it is reasonable to expect a large effect size. In this context, our selection of a medium effect size for mu suppression is conservative: if we do not observe the phenomenon under this assumption, its use in clinical contexts is undermined.

A priori analyses were conducted using G*Power (Faul, Erdfelder, Lang, & Buchner, 2007). A power analysis was carried out to determine the sample size necessary to detect medium effects (*f* = .25, as outlined by Cohen, 1988), with power of .9. As repeated measures ANOVAs typically violate the sphericity assumption, our power analysis considered corrections for sphericity, and was based on the most conservative correction for nonsphericity. This is when the nonsphericity correction (denoted *ɛ*) is equal to 1/1−*m*, where *m* signifies the number of measurements. The anticipated correlation among the repeated measures was set at .6. In previous work, the correlation between occipital and central electrodes has been moderate (see Tangwiriyasakul et al., 2013), and we anticipate that the correlation between different conditions will be moderate to high. We feel this estimation of correlation is justified, especially as the analysis was based on the most conservative nonsphericity corrections possible.

Power analyses demonstrated that to detect a medium effect of condition (videos with hand and object, hand without object and kaleidoscope patterns, and the own movement condition) 52 participants are needed. To detect a medium effect of site (occipital *vs* central) 36 participants are needed. To detect an interaction between these two variables, 61 participants are needed (note that many previous studies have used much smaller sample sizes: (Fan et al., 2010, Muthukumaraswamy and Johnson, 2004a, Muthukumaraswamy and Johnson, 2004b, Muthukumaraswamy et al., 2006, Oberman et al., 2008)). The power of paired *t*-tests to investigate significant effects arising from the ANOVA was also computed; for two-tailed tests, with power of .9, 44 participants are required to detect a medium effect (*d* = .5, Cohen, 1988). The predicted power of these statistical tests with the intended sample size of 61 participants is displayed in Appendix A1. Note that as analyses with the trial-by-trial baseline do not include the own movement condition, which makes the ANOVA for this baseline slightly different. Additional analyses have been conducted for this ANOVA and are detailed in Table 2.

We planned that if data had to be discarded for a participant due too many rejected trials (see the previous section Analysis plan for details on how trials will be rejected and how much data will be allowed to be discarded before a participant is removed from the analysis), further participants would be recruited to ensure the analysis is sufficiently powered. Participants who do not perform the movements during the own movement condition will be reminded by the experimenter to do so, but if the participant continues to not perform the movements they will be excluded from the analysis. If participants need to discontinue the EEG (in the case of sickness) their incomplete recording will be removed from the analysis. A complete record of participants who had to leave the experiment prematurely, or who failed to complete the movement condition, or whose EEG did not produce a sufficient number of good trials, will be kept and the final number of participants that had to be excluded will be reported.

## Appendix C Post-EEG questionnaire

### Questionnaire

Which type of video did you find most interesting to watch?

Which type of video did you find least interesting to watch?

On a scale of 1–5, please rate how engaged you felt you were while watching the blank videos. A score of “1” represents no engagement, while a score of “5” represents a lot of engagement.

On a scale of 1–5, please rate how engaged you felt you were while watching the videos where hands interacted with objects. A score of “1” represents no engagement, while a score of “5” represents a lot of engagement.

On a scale of 1–5, please rate how engaged you felt you were while watching videos where hands interacted without objects. A score of “1” represents no engagement, while a score of “5” represents a lot of engagement.

On a scale of 1–5, please rate how engaged you felt you were while watching the videos of kaleidoscope patterns. A score of “1” represents no engagement, while a score of “5” represents a lot of engagement.

Could you imagine yourself performing the content of the videos? Please rate how hard you think it would be to perform the videos for each video type. A score of 1 would mean that the videos were very easy to perform, while a score of 5 means the videos are hard to perform.

Videos of the hand and the pencil:

Videos of just the hand:

Videos of the kaleidoscope patterns:

Did you notice anything other than the videos of hands or kaleidoscope patterns while you were doing the experiment? If you did, what did you notice?

How many times did you notice these extra stimuli?

## Appendix D Supplementary unregistered analyses

### Responses to post-recording questionnaire

Chi-square tests showed significant effects of condition when participants selected their most interesting [χ^2^ (3) = 61.82, *p* < .001] and least interesting [χ^2^ (3) = 96.05, *p* < .001] condition. One-sample *z* tests comparing these proportions show that the kaleidoscope videos were rated as most engaging significantly more than hand-object videos (*z* = −6.47, *p* < .001), and hand-object videos significantly more than hand-no object videos (*z* = 6.21, *p* < .001). Hand-no object videos were selected as least interesting significantly more than hand-object videos (*z* = −2.53, *p* = .006), and rest more than hand-no object videos (*z* = 13.72, *p* < .001).

One-sample *z* tests also showed that the difference in participant's reports of feeling able to imitate the content of the videos significantly differed between kaleidoscope and hand-object videos (*z* = 20.49, *p* < .001) and hand-no object videos (*z* = 20.89, *p* < .001).

### Gender effects in mu suppression

Independent *t*-tests were conducted comparing males (*N* = 19) and females (*N* = 42) on changes in activity at the central regions during the hand videos. No significant differences were found, for ether video type, for any baseline technique.

## Footnotes

1 These planned comparisons are not corrected, as they were pre-registered. However, readers may wish to take note that for the six comparisons being made for each ANOVA, the Bonferroni corrected alpha cut-off would be .05/6 = .008. However, there are arguably not just six comparisons being made but rather 12 (six for each frequency band for each baseline). Correcting for this number of comparisons, alpha would be .004. Employing these corrected thresholds would mean that a number of the planned comparisons would no longer be significant. For clarity, we list the comparisons affected here. For both the single-long baseline and the between-trials baseline, the difference in beta suppression between kaleidoscope and hand object videos would no longer be significant. For the within-trial baseline, for the mu band, the difference between kaleidoscope and hand-object videos would no longer be significant.
